# The Neuroendocrine-Cardiovascular Axis in Chronic Kidney Disease: The Role of Prolactin and Gonadal Dysfunction

**DOI:** 10.1159/crm/abtag009

**Published:** 2026-09-08

**Authors:** Vincenzo Barbera, Luca Di Lullo, Antonio Bellasi, Lorenzo D’Elia, Annalisa Villani, Paola Peverini, Aldo Franculli

**Affiliations:** a Department of Nephrology and Dialysis, Ospedale L. Parodi Delfino, ASL Roma 5, Colleferro, Italy; b Department of Nephrology and Dialysis, Ospedale dei Castelli, ASL Roma 6, Ariccia, Italy; c Service of Nephrology, Ospedale Regionale di Lugano, Ospedale Civico, Ente Ospedaliero Cantonale (EOC), Lugano, Switzerland; d Faculty of Biomedical Sciences, Università della Svizzera Italiana (USI), Lugano, Switzerland

**Keywords:** Chronic kidney disease, Hyperprolactinemia, Cardiovascular risk, Hypogonadism, Testosterone, Endocrine dysfunction

## Abstract

**Background:**

Chronic kidney disease (CKD) is accompanied by endocrine disturbances, including elevated prolactin and disruption of the hypothalamic-pituitary-gonadal axis. Their reproductive consequences are well recognized, whereas their cardiovascular relevance remains uncertain.

**Summary:**

Reduced renal metabolism, altered dopaminergic regulation, the uremic milieu, inflammation, and comorbidity may increase prolactin and suppress gonadal function. In men, testosterone deficiency becomes more common as kidney function declines. In women, the dominant phenotype is loss of cyclicity, menstrual disturbance, anovulation, and a shortened reproductive lifespan; a single low estradiol value does not adequately characterize this heterogeneous state. Experimental studies identify candidate endothelial, nitric-oxide, inflammatory, thrombotic, and body-composition pathways. Clinical evidence, however, is predominantly observational, is more extensive in men than women, and is inconsistent for prolactin. Non-dialysis CKD and dialysis cohorts are therefore considered separately. Hormone measurement also requires attention to assay method, biological variability, acute illness, medication exposure, menstrual status, and timing relative to dialysis.

**Key Messages:**

Prolactin and gonadal dysfunction are best viewed as components of a hypothesis-generating neuroendocrine-cardiovascular framework rather than established causal cardiovascular risk factors. No evidence supports routine dopamine-agonist treatment of asymptomatic CKD-related hyperprolactinemia or hormone therapy for cardiovascular prevention. Symptom-directed endocrine evaluation, rigorous diagnosis, and CKD-specific prospective and interventional studies are needed.

## Plain Language Summary

Chronic kidney disease (CKD) does more than reduce kidney function. It can also disturb hormones, chemical messengers that control body processes. People with CKD may have higher levels of prolactin, a hormone involved in breast milk production and reproduction. They may also have changes in hormones that control the ovaries or testicles. These problems can affect sexual health, menstrual periods, fertility, muscles, bones, red blood cell production, and quality of life. Researchers are studying whether these hormone changes may also be linked to heart and blood vessel disease, common in CKD. Studies suggest possible links with blood vessels, ongoing irritation in the body, body fat, muscles, and energy use. However, most studies show only that problems occur together, not that one causes another. Findings also differ between people receiving dialysis, a treatment that removes waste and extra fluid from the blood, and those who are not. Evidence in women remains limited. Hormone test results need careful interpretation. Illness, stress, medicines, age, menstrual timing, laboratory methods, and the timing of blood collection around dialysis can all affect the results. Currently, there is no evidence that treating a high prolactin level in a person without symptoms prevents heart disease. The hormones testosterone or estrogen should not be used only to prevent heart disease in CKD. Tests and treatment should be guided by symptoms and a confirmed hormone disorder. Treatments already proven to protect the kidneys and heart remain the priority.

## Introduction

Chronic kidney disease (CKD) is a systemic disorder defined and risk-stratified by cause, glomerular filtration rate (GFR), albuminuria, and, when appropriate, validated kidney-failure risk equations. The 2024 KDIGO guideline places cardiovascular risk reduction within comprehensive CKD care, including blood pressure control, renin-angiotensin system blockade when indicated, sodium-glucose cotransporter-2 inhibition, management of diabetes and dyslipidemia, and attention to lifestyle and complications [1]. Even with such measures, CKD remains strongly associated with cardiovascular events, hospitalization, and death [2, 3].

Endocrine disturbances are part of this systemic phenotype. Elevated prolactin (PRL) and hypothalamic-pituitary-gonadal (HPG) axis dysfunction become more common as kidney function declines and are especially prominent in people receiving dialysis [4, 5]. Their consequences for libido, fertility, menstrual health, bone, erythropoiesis, and body composition are clinically relevant. Whether PRL excess or altered sex hormones independently mediate cardiovascular disease (CVD), rather than reflect kidney dysfunction, inflammation, malnutrition, medication exposure, and comorbidity, is less clear.

The original concept of a neuroendocrine-cardiovascular axis remains useful if it is treated as an organizing model rather than a proven causal chain. In that model, kidney dysfunction perturbs hormone clearance and central regulation; the same uremic and inflammatory environment affects vascular and metabolic biology; and hormonal abnormalities may mark or modify these processes. This distinction matters because a biomarker associated with outcome is not necessarily a therapeutic target.

This narrative review evaluates PRL and gonadal dysfunction across the CKD continuum, separates non-dialysis from dialysis evidence, and distinguishes mechanistic, observational, systematic-review, and interventional data. It also addresses female-specific evidence, practical hormone assessment, and the limits of current treatment data. Table 1 provides a simplified clinical overview, Table 2 maps the evidence by study design and CKD setting, and Table 3 summarizes measurement and interpretation pitfalls.

**Table 1.** Neuroendocrine alterations in CKD: clinical phenotype and evidence-balanced cardiovascular interpretation

Endocrine alteration	Typical CKD context/biochemical pattern	Clinical expression	Cardiovascular interpretation
Elevated prolactin	Reduced renal metabolism/clearance; altered dopaminergic control; medication and stress effects. More frequent and often higher in advanced CKD and dialysis	May be asymptomatic; galactorrhea is uncommon; can contribute to reduced libido, infertility, menstrual disturbance, and secondary hypogonadism	Associated with endothelial measures and outcomes in one CKD/dialysis study, but not independently associated in a later population cohort. Causality and treatment benefit are unproven
Male gonadal dysfunction	Low total and/or free testosterone with low or inappropriately normal LH/FSH is common; primary, secondary, and mixed patterns occur. Acute illness, obesity, SHBG, and medications affect results	Reduced libido, erectile dysfunction, infertility, anemia, low muscle mass, fatigue, bone loss, and frailty; symptoms overlap with uremia	Lower testosterone is associated with inflammation, endothelial dysfunction, and mortality, especially in male cohorts. Residual confounding is likely; CKD-specific cardiovascular treatment trials are absent
Female gonadal/reproductive dysfunction	Disordered GnRH/LH cyclicity and anovulation; estradiol may be low, preserved, or variable depending on age, cycle, menopause, and assay. Amenorrhea during dialysis is not synonymous with menopause	Irregular or absent menses, abnormal uterine bleeding, infertility, sexual dysfunction, and shortened reproductive lifespan	Female cardiovascular evidence is sparse and heterogeneous. Higher estradiol has been associated with all-cause but not consistently cardiovascular mortality in dialysis cohorts; no preventive hormone strategy is established

Cardiovascular statements describe the current level of evidence and should not be interpreted as proof of causality or as treatment recommendations.

CKD, chronic kidney disease; FSH, follicle-stimulating hormone; LH, luteinizing hormone; PRL, prolactin; SHBG, sex-hormone-binding globulin.

**Table 2.** Evidence map for prolactin, gonadal dysfunction, and cardiovascular relevance in CKD

Evidence class	Study	Population/model	Principal finding	Interpretation/limitation
Mechanistic	Reuwer et al., 2011 [6]	Human carotid atherosclerotic plaques	PRL receptor localized to plaque macrophages	Demonstrates local biological capacity; does not show that circulating PRL causes plaque progression or events
Mechanistic	Chang et al., 2016 [7]	Transgenic mouse and vascular experiments	In PRL-overexpressing transgenic mice, increased generation of 16 kDa vasoinhibin inhibited eNOS activity and nitric-oxide production and contributed to increased blood pressure	The transgenic model produced supraphysiological PRL and vasoinhibin exposure and was not a CKD model; the findings cannot be directly extrapolated to moderate CKD-associated hyperprolactinemia
Mechanistic	Wallaschofski et al., 2001 [8]	In vitro human platelet experiments and clinical cohorts with physiological or pathological hyperprolactinemia	PRL enhanced ADP-induced platelet activation in vitro, while hyperprolactinemic pregnant women and patients with pituitary tumors showed greater ADP-induced platelet aggregation	Experimental and small clinical observations outside CKD; they do not establish an increased incidence of thrombotic events ascribable to PRL
Observational – non-dialysis CKD	Carrero et al., 2012 [9]	457 non-dialysis CKD patients	Higher PRL associated with impaired flow-mediated dilation and cardiovascular events; adjusted HR 1.27 per 10 ng/mL	Prospective association but single PRL measure, residual confounding, and no causal intervention
Observational – non-dialysis CKD	Rojhani et al., 2025 [10]	2,005 population-cohort participants; median 19-year follow-up	PRL did not significantly modify the association between CKD and cardiovascular events, and no significant CKD×PRL interaction was detected	PRL was not independently associated with cardiovascular events
Observational – non-dialysis CKD	Yilmaz et al., 2011 [11]	239 men with non-dialysis CKD	Lower total/free testosterone associated with impaired flow-mediated dilation and 72 cardiovascular events	Baseline hormone-FMD associations were cross-sectional, although cardiovascular outcomes were prospectively assessed; residual confounding and reverse causation remain possible
Observational – non-dialysis CKD	Khurana et al., 2014 [12]	2,149 men with CKD stages 3–4	Lowest testosterone quintile showed a borderline adjusted association with mortality	Retrospective, single measurement and substantial comorbidity; effect attenuated with adjustment
Observational – dialysis	Carrero et al., 2012 [9]	173 hemodialysis patients	PRL associated with pulse-wave velocity and with all-cause (HR 1.12) and cardiovascular (HR 1.15) mortality per 10 ng/mL	Observational association; PRL may reflect residual kidney function, inflammation, nutrition, or illness burden
Observational – dialysis	Karakitsos et al., 2006 [13]	100 nondiabetic men receiving hemodialysis	Androgen deficiency associated with greater carotid intima-media thickness and lower flow- and nitrate-mediated dilation	Cross-sectional and male-only; cannot establish directionality
Observational – dialysis	Carrero et al., 2011 [14]	260 men with dialysis-dependent kidney failure	44% had testosterone <10 nmol/L; lower testosterone was independently associated with inflammatory markers and cardiovascular comorbidity	Thresholds are study-specific; inflammation/nutrition can be cause, consequence, or confounder
Observational – dialysis	Gungor et al., 2010 [15]	420 men receiving hemodialysis; 104 deaths	Low testosterone predicted mortality in crude analyses; association attenuated after multivariable adjustment	Illustrates confounding by age, comorbidity, inflammation, and nutrition
Observational – female-specific	Ramesh et al., 2020 [16]	Women initiating hemodialysis	Higher estradiol associated with all-cause and non-CV mortality, but not adjusted CV mortality	Direction opposed a simple “low estrogen is harmful” model; menstrual status and diabetes complicate interpretation
Observational – female-specific	Lau et al., 2023 [17]	427 women undergoing hemodialysis in a prospective Canadian cohort	Estradiol showed a nonlinear, concave association with all-cause mortality, whereas no consistent association was observed with cardiovascular mortality or incident cardiovascular disease	Single baseline hormone measurement, uncertain menopausal status, residual confounding, and limited power for cardiovascular outcomes; the findings do not establish that high estradiol is directly harmful
Observational – female-specific	Gulamhusein et al., 2024 [18]	61 reproductive-aged females with CKD	Testosterone not independently associated with vascular measures; testosterone:estradiol ratio associated with FMD	Small cross-sectional study; ratio finding requires replication
Systematic review/meta-analysis	Oh et al., 2022 [5]	17 CKD studies; 12 enrolled only men	Lower total testosterone associated with CV and all-cause mortality; female evidence insufficient	Heterogeneous thresholds/settings, wide CIs, observational inputs, and no causal inference
Systematic review/meta-analysis	Rytz et al., 2022 [19]	46 studies; 35 meta-analyzed	Menstrual abnormalities common in dialysis; estimated reproductive lifespan 32 years	Substantial heterogeneity; addresses reproductive phenotype rather than cardiovascular mediation
Systematic review/meta-analysis	van der Burgh et al., 2022 [20]	Testosterone/DHEAS and kidney outcomes	Lower testosterone was associated with higher risks of all-cause mortality and cardiovascular events in predominantly male CKD cohorts, whereas evidence for DHEAS and for women was limited	Observational evidence with heterogeneity and limited female data
Interventional	Lo et al., 2017 [21]	Secondary analysis of randomized frequent-hemodialysis trials	More frequent hemodialysis for 1 year did not materially lower PRL	Relevant to dialytic correction of PRL, not dopamine-agonist efficacy or cardiovascular outcomes
Interventional	Ramirez et al., 1985 [22]	10 hemodialysis patients; bromocriptine/endocrine testing	Bromocriptine suppressed PRL but did not normalize broader hypothalamic-pituitary responses	Very small, short, endocrine endpoints only; no cardiovascular outcomes
Interventional	Johansen et al., 2006 [23]	79 hemodialysis patients; factorial randomized trial	Nandrolone increased lean body mass; exercise improved training-specific strength over 12 weeks	Anabolic steroid is not equivalent to physiologic testosterone replacement; no survival/CV outcomes
Interventional – outside CKD	Lincoff et al., 2023 (TRAVERSE) [24]	5,246 men with confirmed hypogonadism and CVD/high risk	Testosterone was noninferior for major adverse CV events; more atrial fibrillation, AKI, and pulmonary embolism were reported	Not a CKD-specific efficacy trial; does not establish benefit or safety in advanced CKD/dialysis

This evidence map separates study design and CKD setting; it is not a formal certainty-of-evidence grade.

AKI, acute kidney injury; CI, confidence interval; CKD, chronic kidney disease; CV, cardiovascular; CVD, cardiovascular disease; eNOS, endothelial nitric-oxide synthase; FMD, flow-mediated dilation; HR, hazard ratio; PRL, prolactin.

**Table 3.** Clinical evaluation of prolactin and gonadal dysfunction in CKD: sampling, interpretation, and pitfalls

Clinical question/analyte	Collection approach	CKD-specific interpretation/pitfall	Suggested next step
When to evaluate	Symptoms/signs or a specific reproductive, bone, pituitary, or sexual-health question	Fatigue, low libido, erectile dysfunction, anemia, depression, and sarcopenia overlap with uremia and comorbidity	Do not use PRL or sex hormones as routine cardiovascular screening biomarkers
Prolactin	Obtain a standardized sample while minimizing venipuncture stress; repeat mild or uncertain elevations. Record pregnancy/lactation, stress, sleep, and medical exposure	CKD may cause moderate elevation. Consider hypothyroidism, liver disease, dopamine antagonists, opioids, verapamil, methyldopa, and estrogen	Test for macroprolactin in asymptomatic patients; request assay dilution when a large pituitary lesion is accompanied by only mild PRL elevation. After excluding physiological, pharmacological, and systemic causes, consider endocrinology referral and pituitary MRI for persistent, disproportionate, or symptomatic hyperprolactinemia
Total testosterone in men	Two separate fasting morning samples using an accurate assay; avoid testing during acute/subacute illness	Age, obesity, diabetes, nutrition, medications, and SHBG alter interpretation. Compatible symptoms or signs plus consistent biochemical deficiency are required	Measure LH/FSH; evaluate reversible causes and pituitary disease as indicated
Free testosterone and SHBG	Use when total testosterone is borderline or SHBG is altered	Direct analog free-testosterone immunoassays are inaccurate	Use equilibrium dialysis or calculate from total testosterone, SHBG, and albumin with a validated equation
LH/FSH	Measure after biochemical hypogonadism is confirmed or when amenorrhea/ovarian dysfunction is being evaluated	High values suggest primary gonadal failure; low or inappropriately normal values suggest central dysfunction. CKD and systemic illness may produce mixed patterns	Add PRL and other pituitary tests when indicated; interpret with clinical context
Female reproductive hormones	Anchor to age, menstrual history, cycle day, pregnancy intention, menopause, and exogenous hormones. Repeat/phase-standardize if management depends on the result	Amenorrhea in dialysis is not synonymous with menopause. Estradiol immunoassays are less precise at low concentrations; one value does not define a chronic phenotype	Exclude pregnancy first. Select TSH, PRL, LH/FSH, estradiol, and targeted androgen testing according to age, menstrual pattern, symptoms, and the suspected diagnosis; involve gynecology or endocrinology when appropriate
Dialysis timing	Use a consistent clock time and a consistent relationship to the dialysis session; a pre-dialysis midweek sample may be practical when serial measurements are planned. Document fasting status, medication timing, and recent illness	Pre-/post-hemodialysis comparisons are affected by volume shifts; residual kidney function, dialysis modality, and metabolic or nutritional factors may influence interpretation	Compare serial results only under similar sampling conditions
Before hormone treatment	Confirm the endocrine diagnosis and a treatment-relevant symptom/indication	Consider fertility goals, hematocrit, prostate evaluation, thromboembolic risk, heart failure and fluid status, sleep apnea, blood pressure, and relevant treatment-specific contraindications	Use multidisciplinary, guideline-based treatment and monitoring. Do not initiate hormone therapy solely to reduce cardiovascular risk

This symptom-directed workflow summarizes general endocrine principles adapted to the CKD setting; it does not establish CKD-specific diagnostic thresholds or treatment indications.

FSH, follicle-stimulating hormone; LH, luteinizing hormone; MRI, magnetic resonance imaging; PRL, prolactin; SHBG, sex-hormone-binding globulin; TSH, thyroid-stimulating hormone.

### Search Strategy and Evidence Selection

For this updated narrative review, PubMed/MEDLINE was searched from database inception through May 2026. The core search was ((“chronic kidney disease” OR CKD OR dialysis OR “kidney failure”) AND (prolactin OR hyperprolactinemia OR hypogonad* OR “gonadal dysfunction” OR testosterone OR estradiol OR estrogen) AND (cardiovascular OR endotheli* OR “arterial stiffness” OR inflamm* OR “oxidative stress” OR mortality)). Targeted searches combined each hormone term with measurement, assay, treatment, dopamine agonist, hormone therapy, menstrual health, or fertility. Reference lists of eligible studies and relevant guidelines were hand-searched.

Peer-reviewed English-language human studies were prioritized. Original cohort studies and trials were preferred for clinical claims; systematic reviews, meta-analyses, and guidelines were used to contextualize consistency, diagnostic practice, and treatment. Experimental and ex vivo studies were included only when they addressed a prespecified candidate pathway. Studies were grouped as mechanistic, observational non-dialysis CKD, observational dialysis, systematic review/meta-analysis, or interventional evidence. Differences in CKD stage, dialysis modality, sex, assay, exposure definition, and outcome were considered during interpretation.

Case reports, non-peer-reviewed material, and articles without relevant endocrine or cardiovascular outcomes were not used to support general claims. Because the aim was a structured narrative synthesis rather than an exhaustive systematic review, no protocol was registered, no formal risk-of-bias instrument was applied, and no new meta-analysis was performed. Candidate records and their evidentiary role were reviewed by the author group and resolved by consensus.

## Physiology of Prolactin and the Hypothalamic-Pituitary-Gonadal Axis

PRL is secreted predominantly by anterior-pituitary lactotrophs. Tonic dopaminergic signaling through D2 receptors restrains secretion, while stress, sleep, estrogen, thyrotropin-releasing hormone, and several medications can increase measured concentrations [25–27]. PRL receptors are widely distributed, and canonical signaling includes JAK2/STAT pathways; biological effects vary by tissue, concentration, exposure duration, and proteolytic processing of PRL into vasoinhibin isoforms [28].

The HPG axis depends on pulsatile hypothalamic gonadotropin-releasing hormone (GnRH), pituitary luteinizing hormone (LH), and follicle-stimulating hormone (FSH), and gonadal steroidogenesis. Sustained pathological hyperprolactinemia can suppress GnRH and contribute to secondary hypogonadism [29]. The relationship is not one-directional – estrogen stimulates lactotroph activity, systemic illness alters gonadotropin pulsatility, and nutrition, adiposity, medications, and age modify sex-steroid concentrations.

Sex steroids have cardiovascular and metabolic actions beyond reproduction. Estrogen-receptor signaling can influence endothelial nitric-oxide synthase (eNOS), vascular smooth-muscle behavior, redox balance, and inflammatory signaling, whereas androgen signaling affects vascular tone, erythropoiesis, muscle protein balance, and body composition [30, 31]. These effects are context-dependent and do not imply that lower concentrations are uniformly harmful or that replacement improves cardiovascular outcomes.

Full-length PRL and proteolytic PRL fragments can have different or even opposing vascular effects. Vasoinhibins inhibit angiogenesis and may alter eNOS signaling, whereas intact PRL can support immune-cell and metabolic signaling. Most clinical assays report immunoreactive total PRL and do not quantify these bioactive fractions. Thus, a higher circulating PRL concentration cannot be translated directly into a single vascular action, especially in CKD, where protease activity and clearance may also be altered [26].

The clinical phenotype also differs by sex and life stage. In men, reduced testicular production and altered sex-hormone-binding globulin (SHBG) may lower total or free testosterone. In premenopausal women, the key abnormality may be failure of cyclic gonadotropin and ovarian responses even when a follicular-phase estradiol value is measurable. After menopause, high gonadotropins and low estradiol represent normal ovarian aging, but CKD-related acyclicity and medication use can obscure the transition. These distinctions are essential when linking a measured hormone to vascular biology.

### CKD as a Neuroendocrine Disorder

The kidney metabolizes and clears several peptide hormones and contributes to the internal milieu in which hypothalamic, pituitary, and gonadal signaling operate. With falling GFR, reduced degradation can increase circulating hormone concentrations, while retained uremic solutes, metabolic acidosis, inflammation, autonomic activation, and protein-energy wasting can perturb secretion or target-tissue response [32–34].

This framework should not convert all endocrine abnormalities into independent disease mediators. CKD severity, diabetes, obesity, frailty, inflammation, medications, and acute illness can simultaneously alter hormone values and cardiovascular prognosis. Accordingly, the same biological variable may be a consequence of kidney failure, a marker of illness burden, a partial mediator, or a combination of these roles. Figure 1 depicts the upstream neuroendocrine disturbances.

**Fig. 1.**
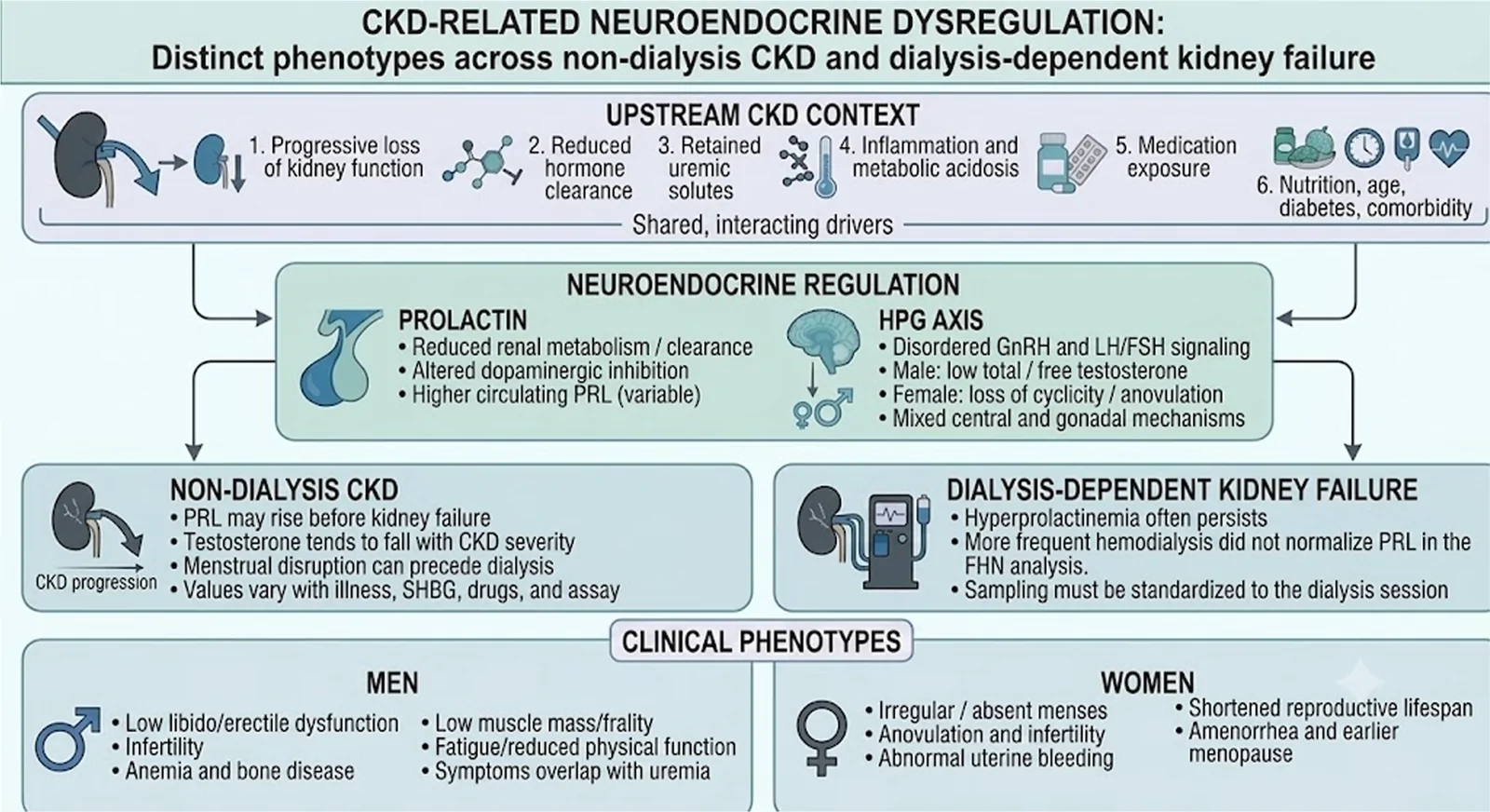
CKD-related neuroendocrine dysregulation across the disease continuum.

Dialysis replaces selected excretory functions but does not reproduce renal endocrine metabolism or fully remove protein-bound and middle-molecule solutes. Residual kidney function, dialysis modality and dose, membrane characteristics, volume status, and treatment vintage may therefore modify hormone concentrations without restoring normal feedback. Successful transplantation often improves PRL and reproductive function, supporting an upstream role for kidney failure, but persistent comorbidity, immunosuppression, and variable graft function prevent uniform recovery [32, 35].

### Hyperprolactinemia and Gonadal Dysfunction in CKD and Dialysis

#### Mechanisms Common to the CKD Continuum

Classic kinetic and stimulation studies indicate that CKD-related PRL elevation reflects both impaired metabolic clearance and altered secretion. Reduced PRL clearance was demonstrated in chronic renal failure, while blunted suppression and stimulation responses supported impaired central regulation [36, 37]. PRL often falls after successful kidney transplantation, although the extent and timing vary [35].

HPG dysfunction is likewise multifactorial. Reduced or disordered GnRH/LH pulsatility, hyperprolactinemia, inflammation, energy deficit, medication effects, and impaired gonadal responsiveness may coexist. In men, this often produces secondary or mixed hypogonadism; in women, loss of cyclicity and anovulation are more accurate descriptors than universal estrogen deficiency [38, 39].

Common CKD medications and conditions can mimic or intensify these patterns. Opioids and glucocorticoids suppress the HPG axis; antipsychotic and antiemetic dopamine antagonists elevate PRL; obesity and insulin resistance lower SHBG; and diabetes, vascular disease, anemia, depression, and neuropathy contribute to sexual dysfunction independently of hormone status. A clinical syndrome should therefore not be attributed to PRL or testosterone without a broader differential diagnosis.

#### Non-Dialysis CKD

PRL may rise before kidney failure, but cross-sectional prevalence estimates vary because studies use different assays, sex-specific reference intervals, medication exclusions, and CKD-stage distributions. Mild-to-moderate elevation should therefore be interpreted in relation to GFR and competing causes rather than automatically attributed to a pituitary lesion or considered a vascular risk marker.

In men, lower total and free testosterone become more frequent with advancing CKD. Symptoms – reduced libido, erectile dysfunction, fatigue, anemia, reduced muscle mass, and depressed mood – overlap substantially with uremia and comorbidity [40, 41]. A low value obtained during acute illness or without symptom concordance is insufficient to diagnose hypogonadism.

The biochemical pattern is not uniform. Gonadotropins may be low, inappropriately normal, or elevated, reflecting central suppression, testicular resistance, or mixed disease. Accumulation of immunoreactive LH fragments can further separate measured concentration from bioactivity. For this reason, study-specific testosterone thresholds are useful for describing cohorts but should not be imported as CKD-specific diagnostic cutoffs [39].

#### Dialysis-Dependent Kidney Failure

In dialysis populations, PRL elevation is common and conventional or more frequent hemodialysis does not reliably normalize pre-dialysis concentrations. In a secondary analysis of the Frequent Hemodialysis Network trials, 1 year of more frequent hemodialysis did not materially reduce PRL [21]. Acute pre-/post-dialysis differences may reflect hemoconcentration, distribution, and assay timing and should not be interpreted as sustained endocrine correction.

Male testosterone deficiency is also highly prevalent. In 260 men with dialysis-dependent kidney failure, 44% had total testosterone below 10 nmol/L, 33% had concentrations of 10–14 nmol/L, and only 23% exceeded 14 nmol/L; lower testosterone correlated inversely with C-reactive protein, interleukin-6, and fibrinogen [14]. These associations support biological interaction with inflammation but cannot determine whether inflammation lowers testosterone, low testosterone increases inflammation, or both.

#### Female-Specific Phenotype

Female reproductive dysfunction in CKD is not adequately summarized as “estrogen deficiency.” Early physiological work in premenopausal women with kidney failure found preserved follicular-phase estradiol and FSH in many participants but loss of cyclicity and impaired positive estradiol feedback, consistent with hypothalamic anovulation [42]. PRL excess may contribute, yet bromocriptine restored ovulation in only one of three treated women in that small study, underscoring multiple mechanisms.

Clinically, menstrual irregularity, amenorrhea, abnormal uterine bleeding, anovulation, infertility, sexual dysfunction, and earlier menopause are reported across advanced CKD and kidney-replacement therapy [43, 44]. A systematic review of 46 studies (35 meta-analyzed) reported menstrual abnormalities in 19–47% of women receiving hemodialysis and 75% of those receiving peritoneal dialysis, with substantial heterogeneity; the estimated reproductive lifespan was 32 years [19]. Kidney transplantation may improve menstrual health, but amenorrhea alone cannot distinguish uremic anovulation from menopause.

This distinction has cardiovascular implications. Cycle phase, age, ovarian reserve, menopause, exogenous hormones, body composition, and diabetes all affect estradiol. Consequently, one concentration, particularly near the lower limits of immunoassays, should not be used to label a woman as estrogen-deficient or to infer cardiovascular risk. Female-specific outcome data remain sparse and, when available, do not support a simple monotonic “lower is worse” model.

Female symptoms also extend beyond amenorrhea. Abnormal uterine bleeding may interact with CKD-related anemia and antithrombotic treatment; vaginal dryness, dyspareunia, and reduced sexual function may be underreported; and fertility can return after intensification of dialysis or transplantation. A careful menstrual and reproductive history is therefore a clinical measurement in its own right, not merely background information for an estradiol assay [19, 43].

## Clinical Evaluation and Hormone-Measurement Pitfalls

No CKD guideline currently recommends universal PRL, testosterone, or estradiol testing for cardiovascular risk prediction. Evaluation is most defensible when prompted by galactorrhea, menstrual disturbance, infertility, sexual dysfunction, gynecomastia, otherwise unexplained bone loss, or symptoms and signs compatible with androgen deficiency. The initial question is clinical – what syndrome is being evaluated, rather than whether an isolated hormone value is outside a laboratory interval.

For PRL, venipuncture stress, pregnancy or lactation, sleep, hypothyroidism, liver disease, chest-wall stimulation, and dopamine-antagonist medications (including antipsychotics and antiemetics), verapamil, methyldopa, estrogens, and opioids should be considered. A mildly elevated result obtained under stress should be repeated under standardized, rested conditions. Macroprolactin testing is appropriate in asymptomatic hyperprolactinemia, and a large pituitary lesion with only modest measured PRL should prompt assay dilution to exclude the hook effect [29].

Persistent PRL elevation that is marked, disproportionate to kidney dysfunction, associated with visual symptoms or headache, or accompanied by otherwise unexplained hypogonadism warrants endocrinology assessment and, when appropriate, pituitary magnetic resonance imaging. CKD is a recognized cause of moderate hyperprolactinemia, but it should not prevent investigation of an alternative cause when the phenotype is discordant.

In men, the Endocrine Society recommends diagnosing hypogonadism only when compatible symptoms or signs coexist with unequivocally and consistently low testosterone. Total testosterone should be measured using an accurate assay on two separate fasting mornings; testing during acute or subacute illness should be avoided. If total testosterone is borderline or SHBG is altered, free testosterone should be measured by equilibrium dialysis or calculated from total testosterone, SHBG, and albumin rather than a direct analog immunoassay [45].

LH and FSH help distinguish primary from secondary or mixed hypogonadism, although altered clearance and chronic illness can complicate interpretation. PRL, iron indices, and other pituitary hormones are added when clinically indicated. Before treatment, fertility goals, hematocrit, prostate risk, sleep apnea, heart failure, recent cardiovascular events, and thrombosis risk require assessment [45].

In women, testing should be anchored to menstrual history, pregnancy possibility and intention, age, cycle day when known, menopause status, and exogenous hormonal use. Pregnancy testing, PRL, thyroid-stimulating hormone, LH, FSH, estradiol, and targeted androgen testing may be appropriate depending on the presentation. Amenorrhea during dialysis is not synonymous with menopause, and low-range estradiol immunoassays have limited precision. Repeated or phase-standardized measurements and gynecologic/endocrine input are preferable when results will change management [43, 44].

For people receiving hemodialysis, serial samples should be collected at a consistent clock time and at the same relation to the dialysis session, commonly before a midweek treatment, and the fasting state, recent illness, and medication timing should be recorded. Pre-/post-dialysis comparisons are vulnerable to volume shifts. Peritoneal dialysis status, residual kidney function, nutritional change, and dialysate glucose exposure may also influence interpretation. Table 3 translates these principles into a practical workflow.

Absolute results should be interpreted using the laboratory’s sex- and method-specific reference interval. PRL immunoassays differ in recognition of macroforms, testosterone methods differ in accuracy at low concentrations, and estradiol immunoassays have limited analytical sensitivity in postmenopausal ranges. When a result is discordant with the clinical picture, confirmation by a more specific method or discussion with the laboratory is preferable to escalating imaging or treatment. Trends are most informative when the same assay platform and sampling conditions are used.

Questionnaires for sexual function, fatigue, and physical performance may help characterize symptoms, but they are not specific tests of endocrine causality. Conversely, a patient-centered benefit – improved sexual function, menstrual health, fertility, bone protection, or anemia – may be clinically meaningful even if cardiovascular benefit is unproven. The indication and intended outcome should be defined before testing and revisited before treatment.

### Prolactin, Sex Hormones, and Cardiovascular Risk outside CKD

Evidence outside CKD helps define plausible pathways but cannot be directly transferred to the uremic setting. Population studies of PRL are inconsistent. One cohort reported positive associations between PRL and all-cause and cardiovascular mortality, whereas Framingham analyses found no association with incident cardiovascular risk factors and another prospective study found no prediction of coronary artery disease [46–48]. Differences in PRL range, sex, menopausal status, single baseline measurements, and exclusion of marked hyperprolactinemia limit comparison.

Small studies of prolactinoma or non-CKD hyperprolactinemia report adverse metabolic or vascular markers and improvement after dopamine-agonist treatment [49, 50]. These before-after observations cannot isolate PRL lowering from recovery of gonadal function, weight change, tumor control, or dopaminergic effects, and they were not designed to test cardiovascular events.

Mechanistic observations are also heterogeneous. PRL receptor expression in macrophages within human carotid plaques establishes local biological capacity, not that circulating PRL causes plaque instability [6]. In a transgenic mouse model, PRL/vasoinhibin signaling reduced eNOS activation and nitric-oxide production and increased blood pressure, but the model involved nonphysiological hepatic overexpression and cannot be equated with moderate CKD-related hyperprolactinemia [7]. PRL enhanced platelet aggregation in one in vitro study, again without proving an in vivo thrombotic effect [8].

For sex steroids, experimental vascular actions and observational associations outside CKD are similarly context dependent. Age, adiposity, diabetes, inflammation, menopause, and underlying illness influence both hormone levels and cardiovascular outcome. These data support candidate mechanisms but not a general cardiovascular indication for testosterone or estrogen treatment.

Testosterone concentration in non-CKD cohorts is strongly related to age, adiposity, diabetes, and chronic illness, while randomized treatment data address selected men with confirmed hypogonadism rather than people with an isolated low value. Likewise, physiological estrogen signaling does not imply that menopausal hormone therapy prevents CVD; vascular response varies with age, time since menopause, formulation, route, and thrombotic risk. These examples caution against converting hormone physiology into a therapeutic claim in CKD.

### The Neuroendocrine-Cardiovascular Axis in CKD

#### Mechanistic Interpretation

The CKD-specific axis is best represented as a network with bidirectional and confounded links (Fig. 2). Inflammation, oxidative stress, metabolic acidosis, energy deficit, and retained solutes can suppress hypothalamic signaling and gonadal steroidogenesis while independently impairing endothelial function and muscle anabolism. Low testosterone or loss of ovarian cyclicity may then modify body composition, erythropoiesis, insulin sensitivity, physical activity, and vascular signaling. Because the upstream drivers are shared, association alone does not establish endocrine mediation.

**Fig. 2.**
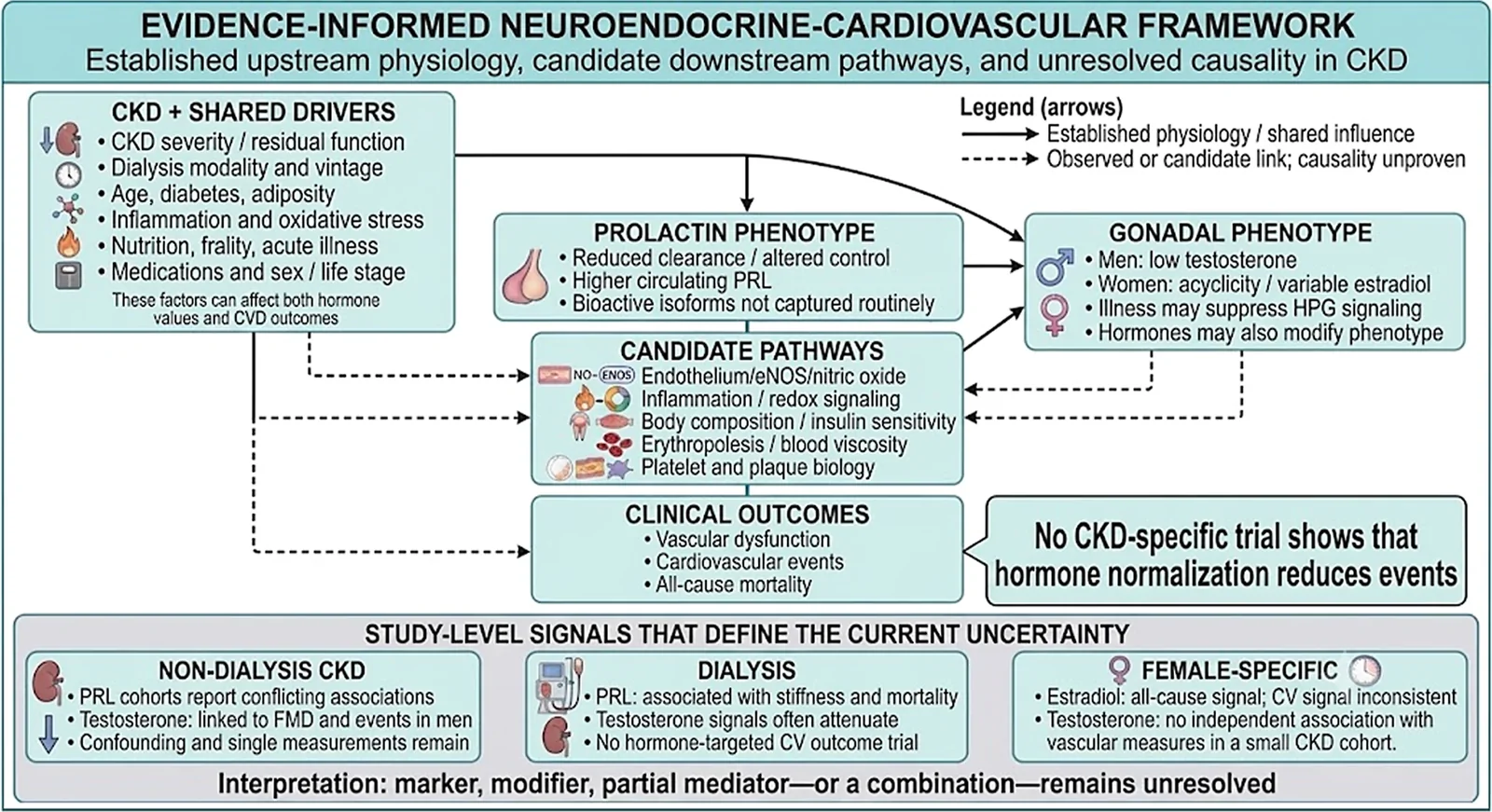
Evidence-informed neuroendocrine-cardiovascular framework in CKD.

The clinical data illustrate this bidirectionality. In dialysis-dependent men, testosterone was inversely related to CRP, interleukin-6, and fibrinogen [14]. This is compatible with cytokine-mediated HPG suppression, with anti-inflammatory effects of androgen signaling, or with low testosterone as part of protein-energy wasting. Experimental eNOS and redox effects provide plausibility, whereas erythrocytosis, fluid retention, and prothrombotic effects of exogenous testosterone demonstrate that biological plausibility is not uniformly protective.

For women, estrogen-receptor signaling can enhance endothelial nitric-oxide bioavailability and influence oxidative and inflammatory pathways [30]. Yet, CKD-related acyclicity, diabetes, menopause, altered body composition, exogenous hormone exposure, and assay limitations complicate serum estradiol as a proxy for tissue signaling. The cardiovascular phenotype should therefore not be reduced to a binary low-estrogen state.

Oxidative stress provides another shared pathway. Uremia reduces nitric-oxide bioavailability and increases reactive oxygen species independently of gonadal status. Experimental estrogen and androgen signaling can modulate antioxidant enzymes, NADPH oxidase, and eNOS coupling, but dose, receptor distribution, and cell type determine direction. Human CKD studies generally measure circulating hormones alongside downstream vascular or inflammatory markers; they do not demonstrate that correcting the hormone reverses the redox defect.

Body composition is a plausible indirect bridge. Low testosterone, acyclicity, inflammation, acidosis, inactivity, and inadequate protein-energy intake can converge on muscle wasting and frailty. Lower muscle mass may reduce physical activity and insulin sensitivity, while frailty predicts hospitalization and mortality. Yet, the same illness burden lowers hormone concentrations, making sarcopenia both a potential mediator and a source of reverse causation. This is why functional outcomes and nutritional covariates are necessary in prospective endocrine studies.

#### Evidence in Non-Dialysis CKD

The principal PRL outcome study included 457 non-dialysis CKD patients and reported associations between higher PRL, impaired flow-mediated dilation, and incident cardiovascular events. After multivariable adjustment, the hazard ratio for cardiovascular events was 1.27 (95% confidence interval [CI], 1.17–1.38) per 10 ng/mL higher PRL [9]. The result is hypothesis-generating – PRL was measured observationally, residual confounding and reverse causation remain possible, and no intervention tested mediation.

A more recent population-based cohort of 2,005 adults followed for a median of 19 years did not find an independent association between PRL and cardiovascular events or a CKD-by-PRL interaction, despite a strong association between CKD and events [10]. Participants generally had milder CKD, marked hyperprolactinemia was excluded, and PRL was measured once. The different populations prevent a direct refutation, but the null result argues against treating PRL as an established independent CKD risk factor.

In 239 men with non-dialysis CKD, lower total and free testosterone correlated with impaired flow-mediated dilation and predicted cardiovascular events during approximately 31 months of follow-up; adjusted hazard ratios per unit increase were 0.83 (95% CI, 0.78–0.88) for total and 0.65 (95% CI, 0.53–0.80) for free testosterone [11]. A larger stages 3–4 CKD cohort found a weaker, borderline association between the lowest testosterone quintile and mortality after adjustment [12]. Both remain vulnerable to illness-related suppression and single-measurement misclassification.

#### Evidence in Dialysis

In the dialysis cohort of the PRL study (173 hemodialysis patients), higher PRL was associated with pulse-wave velocity and with all-cause and cardiovascular mortality: adjusted hazard ratios per 10 ng/mL were 1.12 (95% CI, 1.06–1.17) and 1.15 (95% CI, 1.08–1.21), respectively [9]. The absence of cardiovascular-outcome trials of PRL lowering prevents inference that this association is modifiable.

Testosterone findings are more numerous but still observational. In 100 nondiabetic men receiving hemodialysis, androgen deficiency was associated with greater carotid intima-media thickness and lower flow- and nitrate-mediated dilation [13]. In 260 men with kidney failure, testosterone deficiency was associated with prevalent CVD and mortality [14]. In a 420-man hemodialysis cohort, however, the association between low testosterone and mortality was substantially attenuated after adjustment for age, comorbidity, inflammation, and nutrition [15].

Systematic reviews pool heterogeneous thresholds, dialysis and non-dialysis settings, and mostly male cohorts. One meta-analysis reported that lower total testosterone was associated with cardiovascular mortality (pooled hazard ratio 4.37; 95% CI, 1.40–13.65) and all-cause mortality (1.96; 95% CI, 1.35–2.83), but estimates were imprecise and female data were insufficient [5]. Another synthesis reached a similar direction of association while emphasizing heterogeneity and the absence of causal trials [20].

#### Female Cardiovascular Evidence

Female-specific studies challenge a simple deficiency model. In women initiating hemodialysis, higher – not lower – estradiol was associated with all-cause and non-cardiovascular mortality, particularly among women with diabetes, while no adjusted association with cardiovascular mortality was identified [16]. In a separate prospective hemodialysis cohort, the highest estradiol tertile was associated with higher all-cause mortality in sensitivity analysis, without a consistent cardiovascular association [17].

Among 61 reproductive-aged females with CKD, total testosterone was not independently associated with pulse-wave velocity, augmentation index, flow-mediated dilation, or velocity-time integral; the testosterone-to-estradiol ratio showed a positive association with flow-mediated dilation [18]. The small sample and cross-sectional design preclude outcome inference, but the study demonstrates why female hormonal balance, life stage, and cyclicity require direct study rather than extrapolation from men.

Taken together, the evidence supports an integrated, hypothesis-generating axis. PRL and gonadal abnormalities may identify a neuroendocrine phenotype linked to uremia, inflammation, endothelial dysfunction, and frailty. It does not yet establish that either hormone is an independent causal determinant, that routine measurement improves risk prediction beyond KDIGO-based assessment, or that hormonal normalization reduces cardiovascular events.

#### Therapeutic Perspectives and Future Directions

Management should begin with evidence-based CKD and cardiovascular care rather than endocrine manipulation. Optimization of volume, blood pressure, diabetes, lipids, anemia, nutrition, physical activity, and kidney-protective therapy has a demonstrated clinical rationale; PRL or sex-hormone treatment has not been shown to add cardiovascular benefit in CKD [1].

Routine treatment of asymptomatic CKD-related hyperprolactinemia is not evidence-based and should not be undertaken solely to normalize a laboratory value or reduce cardiovascular risk. The first steps are confirmation, medication and secondary-cause review, assessment for macroprolactin when appropriate, and evaluation for a pituitary disorder when the clinical pattern is disproportionate. Dopamine agonists remain indicated for established prolactinoma and selected symptomatic hyperprolactinemia according to endocrine guidance, with individualized monitoring [29].

CKD-specific dopamine-agonist evidence is minimal. In a small hemodialysis study, bromocriptine suppressed PRL but did not normalize the broader hypothalamic-pituitary response, and no cardiovascular outcomes were studied [22]. Orthostatic symptoms, interactions with antihypertensive therapy, and in high cumulative-dose contexts, fibrotic adverse effects require attention. More frequent hemodialysis did not lower PRL in the Frequent Hemodialysis Network analysis [21].

Testosterone therapy should likewise follow diagnostic rigor rather than a single low value. In symptomatic men with confirmed hypogonadism, treatment may be considered after evaluation of cause, fertility goals, prostate risk, hematocrit, sleep apnea, fluid status, heart failure, and thrombotic risk. Monitoring should follow the Endocrine Society guideline [45]. CKD-specific trials have not established cardiovascular benefit, and testosterone should not be prescribed for cardiovascular prevention.

The TRAVERSE trial in 5,246 men with symptomatic, confirmed hypogonadism and preexisting or high cardiovascular risk found testosterone gel noninferior to placebo for major adverse cardiovascular events (hazard ratio 0.96; 95% CI, 0.78–1.17) but reported more atrial fibrillation, acute kidney injury, and pulmonary embolism in the testosterone group [24]. This was not a CKD-specific efficacy trial and cannot establish benefit or safety in advanced CKD or dialysis.

Anabolic-steroid studies in dialysis should not be equated with physiological testosterone replacement. In a 79-patient randomized trial, nandrolone increased lean body mass over 12 weeks, while resistance exercise improved training-specific strength; survival and cardiovascular outcomes were not tested [23]. These findings support research on physical function, not routine endocrine treatment.

Kidney transplantation is the intervention most consistently associated with improvement in CKD-related PRL and reproductive abnormalities, but it is not an endocrine treatment and is subject to selection, graft function, and immunosuppressive effects [35, 36]. Dialysis intensification may benefit volume, solute control, and reproductive health in selected patients, but the randomized frequent-hemodialysis analysis did not demonstrate PRL normalization [21]. These observations argue against using PRL change as a surrogate for dialysis adequacy.

For women, neither estradiol measurement nor menopausal hormone therapy has a validated role in CKD cardiovascular prevention. Treatment for menopausal, gynecologic, bone, or reproductive indications should be individualized with nephrology and gynecology/endocrinology input, considering age, route, uterine status, thrombosis, blood pressure, and dialysis-access risk. Female-specific trials are a priority.

Future studies should prespecify CKD stage and dialysis modality; use repeated, standardized hormone measurements and validated assays; enroll adequate numbers of women; account for cycle and menopause; and separate prediction from mediation. Trials should target symptomatic, biochemically confirmed phenotypes and measure patient-reported function, vascular intermediate outcomes, safety, and when feasible, cardiovascular events. Mendelian-randomization or causal-mediation analyses may help prioritize interventions but cannot replace trials.

#### Limitations of Current Evidence

Most evidence is observational, often single-center, and based on one hormone measurement. Definitions of hyperprolactinemia and hypogonadism, assay methods, sampling time, CKD stage, dialysis modality, covariate adjustment, and outcome ascertainment vary. Confounding by age, diabetes, obesity, inflammation, malnutrition, medication exposure, and illness severity is substantial; reverse causation is plausible. Combining non-dialysis and dialysis populations can obscure important biological and clinical differences.

Women are underrepresented, and menstrual phase, menopause, exogenous hormones, and assay performance are frequently incompletely characterized. Mechanistic studies often use supraphysiological exposure or non-CKD models. Finally, this was a structured narrative review without protocol registration or formal risk-of-bias scoring; the evidence table is an interpretive map, not a graded clinical-practice recommendation.

## Conclusion

CKD-related hyperprolactinemia and HPG-axis dysfunction are common and clinically relevant, but their cardiovascular meaning requires restraint. Mechanistic studies identify plausible endothelial, inflammatory, redox, thrombotic, and body-composition pathways; clinical studies show associations that vary by CKD setting, sex, and study design.

The most defensible interpretation is a hypothesis-generating neuroendocrine-cardiovascular network in which endocrine abnormalities may be markers, modifiers, or partial mediators of a shared uremic phenotype. They are not established independent causal risk factors, and current evidence does not support hormone-based cardiovascular screening or treatment.

Clinical care should focus on symptom-directed, methodologically sound endocrine evaluation and on validated CKD and cardiovascular therapies. Routine treatment of asymptomatic CKD-related hyperprolactinemia is not evidence based. Prospective, sex-inclusive, CKD stage-specific studies and appropriately powered interventional trials are required before endocrine correction can be considered a cardiovascular strategy.

## Conflict of Interest Statement

Dr. Luca Di Lullo and Dr. Antonio Bellasi were members of the journal’s Editorial Board at the time of submission. The remaining authors have no conflicts of interest to declare.

## Funding Sources

This research received no specific grant from any funding agency in the public, commercial, or not-for-profit sectors.

## Author Contributions

V.B., L.D.L., and A.F. contributed to the conceptualization and design of the review. V.B. and A.F. drafted the initial manuscript. L.D.E., A.V., and P.P. conducted the literature search and summarized the evidence. A.B. and L.D.L. critically revised the work for important intellectual content and provided supervision. All authors have read, reviewed, and approved the final version of the manuscript.
